# Magnetic Resonance Imaging of Contrast-Induced Acute Renal Injury and Related Pathological Alterations In Vivo

**DOI:** 10.1155/2022/6984200

**Published:** 2022-02-26

**Authors:** Yanfei Li, Dafa Shi, Haoran Zhang, Xiang Yao, Ke Ren

**Affiliations:** Department of Radiology, Xiang'an Hospital of Xiamen University, School of Medicine, Xiamen University, Xiamen, 361102 Fujian, China

## Abstract

**Background:**

The definitive mechanisms of CI-AKI include contrast medium (CM) nephrotoxicity and CM disturbances in renal blood flow, but how the immune system responds to CM has rarely been mentioned in previous studies, and different cell death pathways have not been clearly distinguished.

**Aim:**

To confirm whether MRI detect early CI-AKI and to investigate whether immunity-related responses, pyroptosis, and mitophagy participate in contrast-induced acute renal injury (CI-AKI).

**Methods:**

C57BL/6 mice with CI-AKI were established by tail vein injection of iodixanol 320. Magnetic resonance imaging of 9.4 T scanner and microscopic appearance of renal H&E staining were tools to test the occurrence of CI-AKI at different times. Immunohistochemistry and NGAL were used to examine the immune responses in the kidneys with CI-AKI. Transmission electron microscopy and western blot methods were used to distinguish various cell death pathways in CI-AKI. *Key Results*. The densitometry of T2WI, DTI, and BOLD presents CI-AKI in a regular way. The microscopic appearance presents the strongest renal damage in CI-AKI mice that existed between 12 h (*P* < 0.0001) and 24 h (*P* < 0.05) after contrast medium (CM) injection. Strong correlation may exist between MRI densitometry (T2WI, DTI, and BOLD) and pathology. Neutrophil and macrophage chemotaxis occurred in CI-AKI, and we observed that Ly6G was the strongest at 48 h (*P* < 0.0001). Pyroptosis (Nlrp3/caspase-1, *P* < 0.05), mitophagy (BNIP/Nix, *P* < 0.05), and apoptosis (Bax, *P* < 0.05) occurred in CI-AKI.

**Conclusions:**

fMRI can detect early CI-AKI immediately after CM injection. NLRP3 inflammasomes are involved in CI-AKI, and mitophagy may play a role in mitigating kidney injury. The mitochondrion is one of the key organelles in the tubular epithelium implicated in CI-AKI.

## 1. Introduction

Contrast-induced nephropathy (CIN) was defined by the Contrast Media Safety Committee (CMSC) of the European Society of Urogenital Radiology (ESUR) in 2011 [1]. The term for acute kidney injury associated with contrast medium (CM) administration when no control population is available is postcontrast acute kidney injury (PC-AKI), and the term contrast-induced acute kidney injury (CI-AKI) should be used only when compared with a control, allowing CM to be shown as the cause of the acute kidney injury (AKI) [2]. In clinical practice, PC-AKI and CI-AKI should be defined as an increase in creatinine (sCr) of ≥0.3 mg/dl or of ≥1.5-1.9 times the baseline in the 48-72 h following CM administration [2]. The definitive mechanisms of CI-AKI may include CM nephrotoxicity, such as endothelial damage resulting from oxidative stress [3], and CM disturbances in renal blood flow [4]. However, many potential renal responses to CM need to be interpreted. How the immune system responds to CM has seldom been mentioned in previous studies, and the different cell damage/death pathways have not been clearly distinguished. In this study, we first established a mouse model of CI-AKI at first. Functional magnetic resonance imaging (fMRI) and hematoxylin and eosin (H&E) staining were used to test and predict CI-AKI. Immunological histological chemistry (IHC) of Ly6G and Nlrp3, transmission electron microscopy (TEM), and western blot were used to identify the potential mechanisms of CI-AKI.

## 2. Methods

### 2.1. Animal Model and MRI Imaging

**Figure 1:**
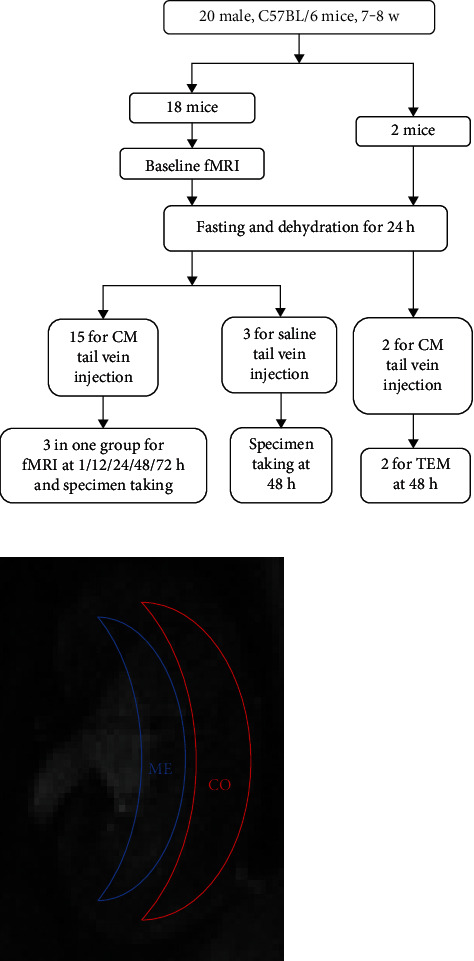
Overall experiment design and ROIs. All the mice were 7-8 w male C57BL/6. Baseline fMRI was performed on 15 randomly chosen mice. After a 24-hour fasting and dehydration, CM injection was performed on these 15 mice and saline injection was performed on 3 randomly chosen control mice. At five different times, fMRI was performed on every 3 randomly chosen mice of CM injection and the kidneys and blood were taken for further study. Another 2 mice were given CM injection the same as before, and the kidneys were taken at 48 h for TEM. Two ROIs were measured, the red region CO represents the cortex, and the blue region ME represents the medulla. fMRI: functional magnetic resonance imaging; CM: contrast media; TEM: transmission electron microscope.

We chose 7-8 w old male C57BL/6 mice as model candidates (a total of 18 mice were used, 15 for experimental groups and 3 as controls). All the mice were provided by Xiamen University Laboratory Animal Center and raised under 12 h/12 h day/night cycles, a temperature of 22 ± 2°C, and a relative humidity of 50-70%. Mouse kidney baseline fMRI was performed after anesthetization with isoflurane (flow rate: 300-500 ml/min, induction concentration: 3-4%, and maintenance concentration: 1-1.5%). All MRI experiments were performed on a horizontal bore 9.4 T scanner operating on a Bruker Avance platform (Bruker 9.4 T MicroMRI, BioSpec 94/20USR) over the coronal renal region in a prone position, head-in first. Parameters and sequences are shown in Table 1. After fasting and dehydration for 24 h, mice were injected with CM (iodixanol 320) at a dose of 4 g (I)/kg or an equal volume of saline through the tail vein [5]. After that and at the following different times, 1, 12, 24, 48, and 72 h, 3 mice in a group underwent fMRI in the same way as above. All the images were obtained regions of interest (ROIs) and performed densitometry measurements by ImageJ software. The radiologists were blinded to this performance. The overall experimental design and ROIs are shown in Figure 1.

### 2.2. Sample Collecting

Soon after obtaining the fMRI images, blood was collected from the eyeballs removed from mice in separate gel coagulation-promoting tubes and then centrifuged at 4°C and 4,000 rpm for 10 min. The serum was collected and stored at -80°C. Kidneys were removed from the renal hilus and fixed for paraffin sectioning in 4% paraformaldehyde for 48 h. All mice were euthanized via intraperitoneal injection of 0.3% sodium pentobarbital (8 ml/kg), according to the guidelines of the Institutional Animal Care and Use Committee (IACUC) of Xiamen University.

### 2.3. Hematoxylin and Eosin Staining

The kidneys were dehydrated with ethanol and dimethylbenzene, and 4 *μ*m paraffin sections were obtained. H&E staining was performed according to the kit instructions (H&E staining kit, C0105S, Beyotime, China). The kidney was histologically scored to evaluate renal damage according to tubular desquamation, necrosis, atrophy, cytoplasmic vacuoles, and interstitial infiltration. The renal injuries were graded under a microscope according to the following score: 0, normal kidney tissue; 1, minimal injury (0-5%); 2, moderate injury (5-25%); 3, intermediate injury (25-75%); and 4, severe injury (75-100%) [6]. Two independent pathologists who were blinded to the experiment completed the assignment according to the criteria.

### 2.4. Immunohistochemistry

After deparaffination, antigen retrieval was performed in citrate antigen retrieval solution. Endogenous peroxidase activity was blocked with 3% H_2_O_2_. The sections were then incubated with primary antibodies polyclonal anti-Ly6G (1 : 100, orb322983, Biorbyt, UK) and anti-Nlrp3 (1 : 100, NBPZ-12446, Novus Biological, USA) at 4°C overnight and with secondary antibody-labeled HRP for 30 min. Diaminobenzidine (DAB) was used to reveal the expression of Ly6G and Nlrp3. The intensity of Ly6G and Nlrp3 staining was evaluated by the *H*-score, which was calculated by adding the multiplication of the different staining intensities (SI) in 4 gradations with each percentage of positive cells. The *H*-score could range from 0 to 300 points; a sample with an *H* − score ≥ 50 points was considered positive. *H* − score = [1 × (%cells 1+) + 2 × (%cells 2+) + 3 × (%cells 3+)] [7]. Two independent pathologists who were blinded to the experiment completed the assignment according to the criteria.

### 2.5. Serum ELISA

The serum concentration of neutrophil gelatinase-associated lipocalin (NGAL) was tested with a serum enzyme-linked immunosorbent assay (ELISA) at Wuhan Servicebio Technology.

### 2.6. Protein Extraction and Western Blot

The kidney was cut into pieces, and the debris was homogenized with ultrasonication. The sample was then centrifuged, and the supernatant was collected. SDS-loading buffer was added to denature the protein. Approximately 10 *μ*l of sample was added to perform gel electrophoresis. Thereafter, the samples were transferred onto a nitrocellulose filter membrane. Nonspecific binding sites were blocked with skim milk powder at room temperature and then incubated with primary antibodies against Nlrp3 (1 : 1000, NBPZ-12446, Novus Biological, USA), caspase-1 (1 : 1000, ab179515A, Abcam, UK), caspase-8 (1 : 1000, #4790, CST, USA), GAPDH (1 : 1000, 10494-1-AP, Proteintech, China), BAX (1 : 1000, 50599-2-Ig, Proteintech, China), Bcl-2 (1 : 1000, ab196495, Abcam, UK), Cytochrome C (1 : 1000, #11940, CST, USA), NF-*κ*B (1 : 1000, #8242, CST, USA), and BNIP3L/Nix (1 : 1000, #12396, CST, USA) at 4°C overnight. The membrane was incubated with horseradish peroxidase- (HRP-) labeled conjugated secondary antibodies at room temperature. The immunostained protein bands were visualized through a chemiluminescence system. The relative densitometry was analyzed by ImageJ software.

### 2.7. Transmission Electron Microscopy

Another two mice were treated as described above for CI-AKI establishment. Then, the kidneys were collected and fixed, dehydrated, embedded, sectioned, and stained. Briefly, fresh tissue blocks were cut and harvested quickly within 3 min. Tissues were blocked from light and incubated in 1% OsO_4_ postfixing at room temperature for 2 h. After removing the OsO_4_, the tissues were rinsed. The embedding models with resin and samples were moved into an oven for polymerization for more than 48 h. Then, the resin blocks were removed from the embedding models for standby application at room temperature. The resin blocks were cut to 60-80 nm and subjected to 2% uranium acetate saturated alcohol solution staining and 2.6% lead citrate staining. Typical images were captured by a Hitachi HT7700 electron microscope.

### 2.8. Statistical and Analysis

All the values were calculated and analyzed with GraphPad Prism 5. The histological score, NGAL concentration, and relative densitometry data between groups were analyzed with one-way ANOVA. Spearman correlation analysis was used to study the linkage between MRI densitometry and pathology. *P* < 0.05 was considered statistically significant.

## 3. Results

### 3.1. Renal fMRI

**Figure 2:**
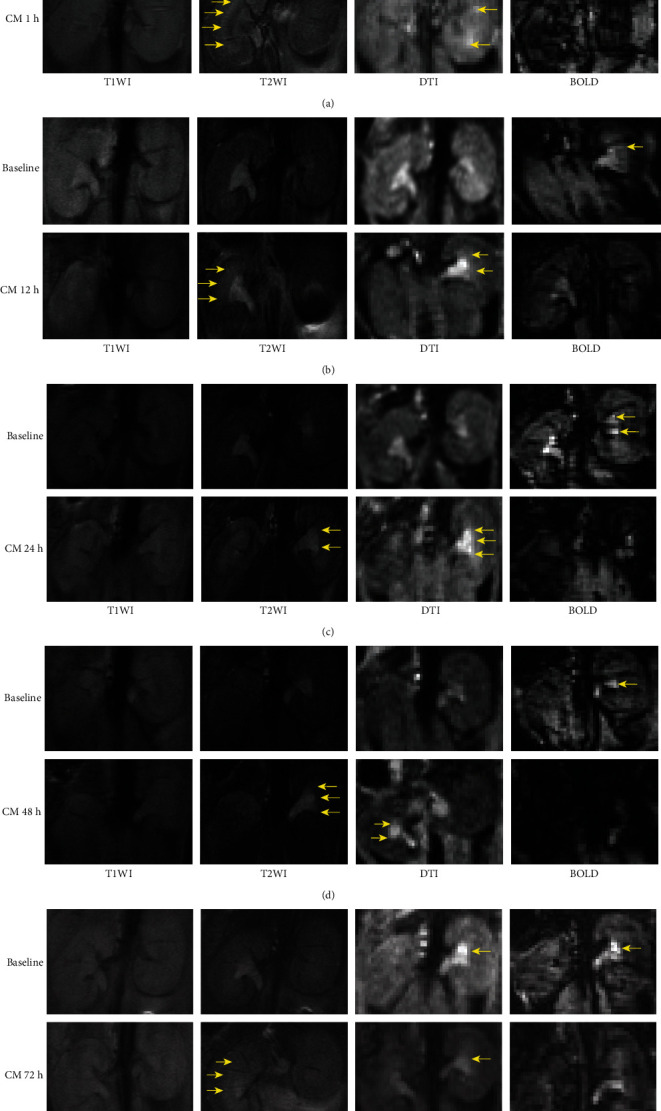
Representative kidney MRI imaging (a–e). The arrows show the alterations related to injection of CM. No obvious alterations present on T1WI. Significant enhancing signal alterations present on T2WI and DTI. Some signal-associated oxygen contents disappear on BOLD imaging after CM administration. CM: contrast media; DTI: diffusion tensor imaging; BOLD: blood oxygen level-dependent imaging; T1WI: T1-weighted image; T2WI: T2-weighted image.

**Figure 3:**
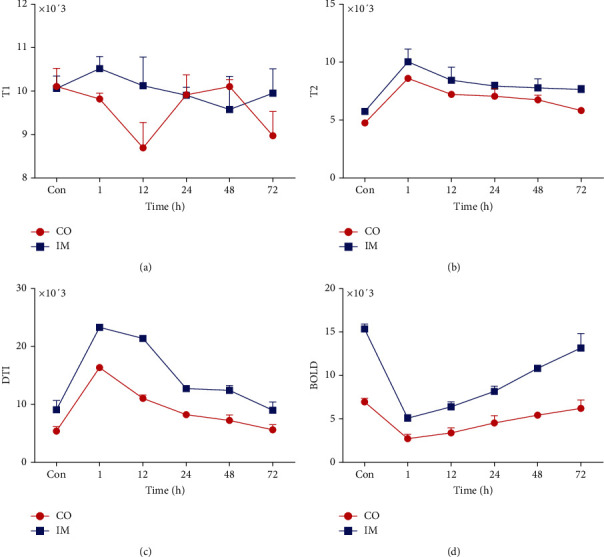
The densitometry of MRI at different times after CM injection. (a) T1WI; (b) T2WI; (c) DTI; (d) BOLD. CO: cortex; IM: inner medulla; CM: contrast media; DTI: diffusion tensor imaging; BOLD: blood oxygen level-dependent imaging; T1WI: T1-weighted image; T2WI: T2-weighted image.

Representative fMRI images are shown in Figure 2. After CM injection, renal hypoxia was detected with blood oxygen level-dependent (BOLD) imaging via a decreased oxygen level and a decreased signal. On diffusion tensor imaging (DTI), water molecules with restricted diffusion and enhanced signals were detected. T2-weighted imaging (T2WI) showed enhanced signal on the boundary between the cortex and medulla, and T1-weighted imaging (T1WI) showed no signal changes. In mouse A, 1 h after CM administration, the oxygen content and diffusion began decreasing relative to baseline; on T2WI, a clear “boundary” appeared between the cortex and medulla, as indicated by the arrow. Mouse B underwent fMRI after CM injection at 12 h, and similar changes to mouse A were observed. In mice C and D, 24 h and 48 h after CM administration, BOLD and DTI signals presented with similar characteristics as those of mouse A and mouse B, but the enhanced signal on T2WI appeared closer to that of the renal pelvis. In mouse E, 72 h after administration, the signal changes relative to baseline on BOLD and DTI images were not as clear as those of mice A, B, C, and D, but T2WI still showed an enhanced signal.  Figure 3 shows the densitometry of MRI at different times after CM injection. The graphs of T2WI and DTI densitometry present an increase within 1 h after CM injection and then decrease gradually. The graph of BOLD densitometry presents a decrease within 1 h after CM injection and then increases gradually. However, the graph of T1WI displays irregularly.

### 3.2. Microscopic Appearance of the Kidney

**Figure 4:**
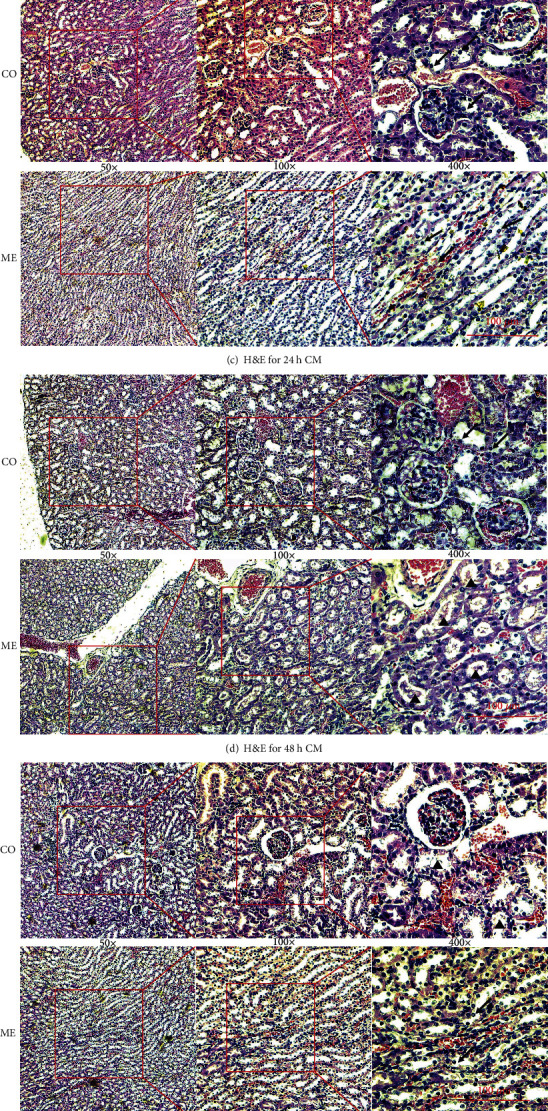
Renal microscopic H&E images. The upper row of each group presents the renal cortex (CO) and the second row presents the renal medulla (ME). (a) CM for 1 h, swollen and shedding tubular epithelium (arrows). (b) CM for 12 h, cytoplasmic vacuoles (stars) in tubular epithelium and shedding vacuolated cells (stars) budged into the lumen. (c) CM for 24 h, nuclei desquamated from tubular epithelium (arrows). (d) CM for 48 h, cast appeared (triangles). (e) CM for 72 h, cytoplasmic vacuoles (stars) and cast (triangles) disappeared gradually. (f) Saline for control, some shedding tubular epithelium (arrows). (g) Histological score of the kidney shows renal injury began at 1 h and recovered at 24 h after CM injection; ^∗^*P* < 0.05, ^∗∗^*P* < 0.005, and ^∗∗∗^*P* < 0.0001. Original magnification: 50x, 100x, and 400x.

Figures 4(a)–4(f) show the representative microscopic images of the kidney. At 1 h, tubular epithelial cells became swollen and started shedding slightly, and nuclei became desquamated from tubular epithelial cells. At 12 h, cytoplasmic vacuoles began appearing in the tubular epithelial cells. Shed vacuolated cells budged into the tubular lumen. At 24 h, more nuclei became desquamated from the tubular epithelial cells. At 48 h, a cast appeared in the renal tubules. At 72 h, the cytoplasmic vacuoles and casts disappeared from the renal tubules. The control group only had some shedding tubular epithelium.

The histological score in Figure 4(g) represented the injuries that appeared at 1 h (*P* < 0.05) after CM injection and gradually recovered after 24 h (*P* < 0.05). A constant recovery was maintained until 72 h (*P* < 0.0001).

### 3.3. Correlation between MRI Densitometry and Pathology

**Figure 5:**
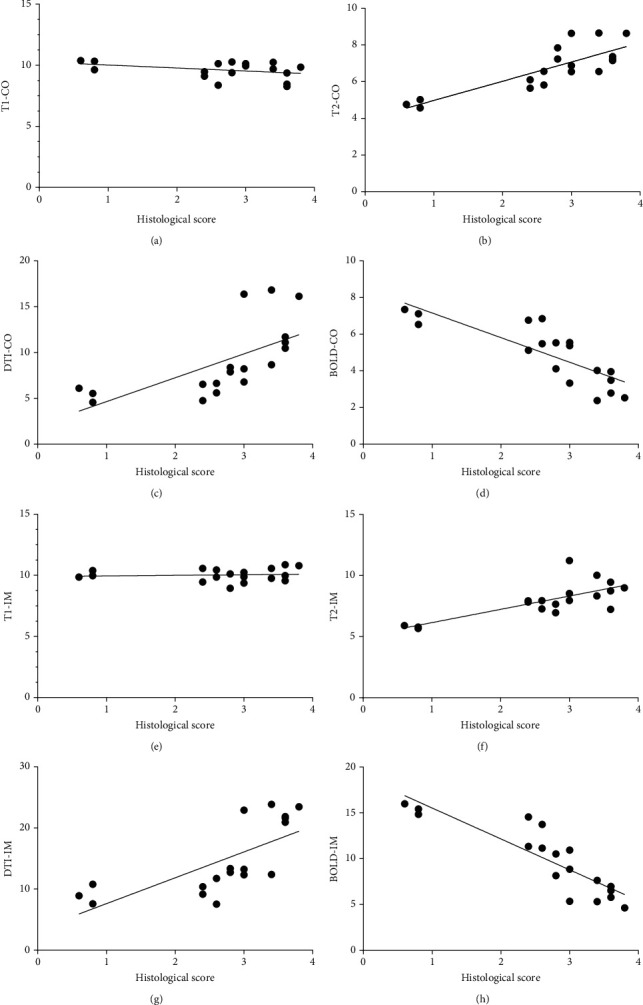
Correlation between MRI densitometry and pathology: (a) T1-CO vs. histology: *r* = −0.3565 (*P* = 0.1465); (b) T2-CO vs. histology: *r* = 0.8217 (*P* < 0.0001); (c) DTI-CO vs. histology: *r* = 0.6495 (*P* = 0.0035); (d) BOLD-CO vs. histology: *r* = −0.8156 (*P* < 0.0001); (e) T1-IM vs. histology: *r* = 0.09259 (*P* = 0.7148); (f) T2-IM vs. histology: *r* = 0.7451 (*P* = 0.0004); (g) DTI-IM vs. histology: *r* = 0.71 (*P* = 0.001); (h) BOLD-IM vs. histology: *r* = −0.8744 (*P* < 0.0001). CO: cortex; IM: inner medulla; CM: contrast media; DTI: diffusion tensor imaging; BOLD: blood oxygen level-dependent imaging; T1WI: T1-weighted image; T2WI: T2-weighted image.

No correlation was calculated between T1WI densitometry and histological score. A strong positive correlation was calculated between T2WI densitometry and histological score. Another strong positive correlation was calculated between DTI densitometry and histological score. A strong negative correlation was calculated between BOLD densitometry and histological score. These results are shown in Figure 5: (a) T1-CO vs. histology: *r* = −0.3565 (*P* = 0.1465), (b) T2-CO vs. histology: *r* = 0.8217 (*P* < 0.0001), (c) DTI-CO vs. histology: *r* = 0.6495 (*P* = 0.0035), (d) BOLD-CO vs. histology: *r* = −0.8156 (*P* < 0.0001), (e) T1-IM vs. histology: *r* = 0.09259 (*P* = 0.7148), (f) T2-IM vs. histology: *r* = 0.7451 (*P* = 0.0004), (g) DTI-IM vs. histology: *r* = 0.71 (*P* = 0.001), and (h) BOLD-IM vs. histology: *r* = −0.8744 (*P* < 0.0001).

### 3.4. Renal Inflammation in CI-AKI

**Figure 6:**
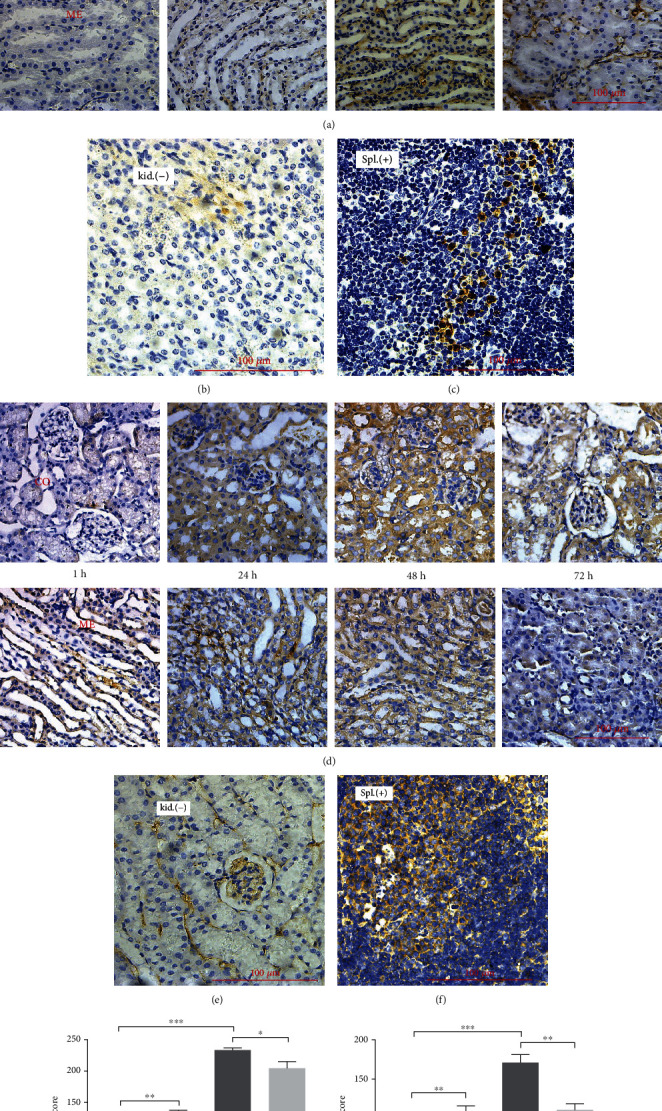
Renal microscopic IHC images. (a, d) IHC of Ly6G and Nlrp3 in the kidney after CM injection. The upper row of this group of pictures shows the renal cortex (CO) and the second row the renal medulla (ME). From left to right, each one of the four pictures indicates 1 h, 24 h, 48 h, and 72 h after CM injection. (b, e) Ly6G and Nlrp3 without primary antibody in the kidney as negative control kid (-). (c, f) Ly6G and Nlrp3 in the spleen as positive control Spl (+). (g, h) Are the *H*-score for IHC of Ly6G and Nlrp3; ^∗^*P* < 0.05, ^∗∗^*P* < 0.005, and  ^∗∗∗^*P* < 0.0001. Original magnification: 400x.

Renal IHC of Ly6G is shown in Figure 6(a). Ly6G is an antigen expressed on the neutrophils of mice. Ly6G expression was stronger in the glomeruli than in medulla tubular epithelial cells at 1 h, but at 48 h, these expression levels were reversed. This may mean that neutrophils slowly extravasated from the vessels and capillary to the renal mesenchyme around the tubular epithelium.  Figure 6(g) shows that the strongest staining of Ly6G appeared at 48 h (*P* < 0.0001), which indicates that the neutrophils might act as representative inflammatory cells in CI-AKI to clear endogenous damaged cellular components. At 72 h, the staining became weaker (*P* < 0.05), which implies waning inflammation and renal relief.

Renal IHC of Nlrp3 is shown in Figure 6(d). The noncanonical nucleotide oligomerization domain-like receptor pyrin 3 (NLRP3) inflammasome is expressed in tubular cells, and the canonical NLRP3 inflammasome is expressed in myeloid cells. Compared with 1 h, Nlrp3 expression was much stronger at 24 h (*P* < 0.005) and remained stronger at 48 h (*P* < 0.0001) but weakened in 72 h (*P* < 0.005). It was difficult to differentiate the two origins of NLRP3 inflammasome. The Nlrp3 score in Figure 6(h) was similar to the Ly6G score, which also indicates that the strongest inflammatory response appeared at 48 h after CM injection. Figures 6(b) and 6(e) are the kidney negative controls without primary antibody of Ly6G and Nlrp3. Figures 6(c) and 6(f) are the spleen positive controls for Ly6G and Nlrp3.

### 3.5. Proteins Related to CI-AKI

**Figure 7:**
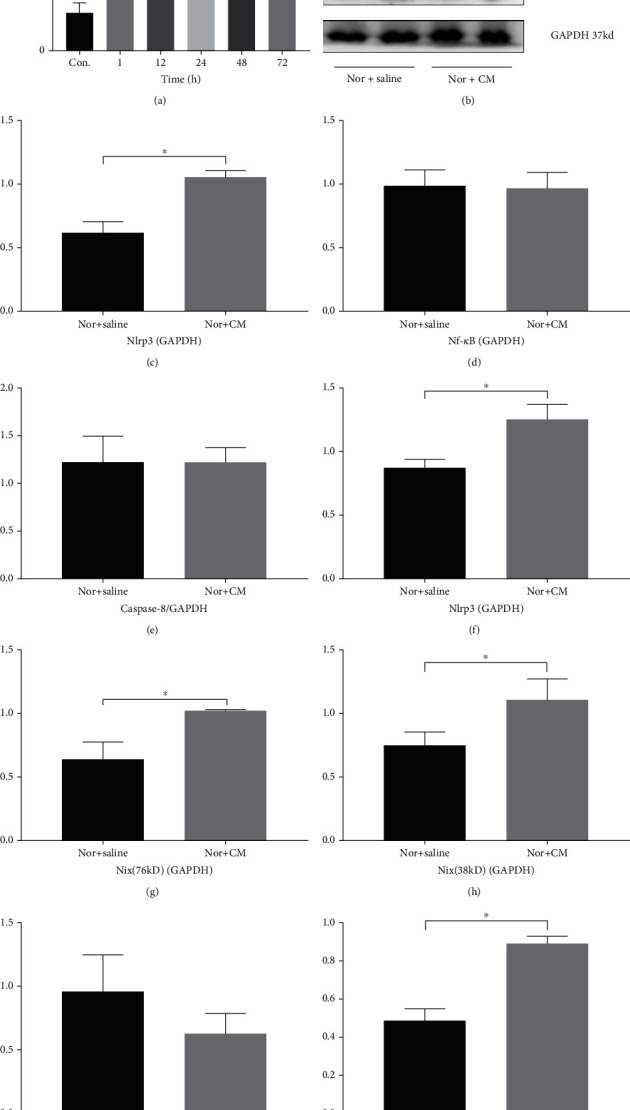
Protein related to CI-AKI. (a) Serum NGAL. A dramatic increasing of NGAL presents at 24 h after CM injection. (b) Western blotting of the kidney. After administration of CM for 48 h, the expression of Nlrp3 and pro-caspase-1 got a marked increase. NF-*κ*B, caspase-8, CytoC, and BAX got a moderate increase. On the contrary, Bcl-2 got a moderate decrease. BNIP3L/Nix, which is related to autophagy and mitophagy, also got an obvious increase. GAPDH is internal control. (c–k) Relative densitometry analysis; ^∗^*P* < 0.05 and ^∗∗^*P* < 0.005. Nlrp3: nucleotide oligomerization domain-like receptor pyrin 3; NF-*κ*B: nuclear factor kappa-B; cas8: caspase8; pro-cas1: pro-caspase1; BNIP3L/Nix: Bcl2 interacting protein3/Nip3-like protein; Bcl2: B-cell lymphoma-2; BAX: Bcl2-associated X; CytoC: cytochrome C; GAPDH: glyceraldehyde-3-phosphage dehydrogenase; nor: normal; CM: contrast media; nor: normal.

In Figure 7(a), the amount of NGAL increased 1 h after CM injection, and a dramatic difference between the control group and CI-AKI groups appeared at 24 h (*P* < 0.005). No renal injury seemed to relieve within 72 h if NGAL was taken as a regular AKI marker. The NLRP3 inflammasome and caspase-1 are core components of pyroptosis. In Figure 7(b), proteins related to pyroptosis, mitophagy, and apoptosis are represented by western blot. The expression of Nlrp3 and pro-caspase-1 was markedly increased in our experiment. NF-*κ*B, caspase-8, CytoC, and BAX are apoptosis-associated proteins and showed a moderate increase. In contrast, the antiapoptosis protein, Bcl-2, showed a moderate decrease. Bcl2 interacting protein3/Nip3-like protein (BNIP3L/Nix), which is related to autophagy and mitophagy, was obviously increased. GAPDH is internal control. Figures 7(c)–7(k) show a relative densitometry analysis, which further shows that pyroptosis and mitophagy play an important role in CI-AKI (Nlrp3, *P* < 0.05; caspase-1, *P* < 0.05; BNIP/Nix, *P* < 0.05).

### 3.6. Mitophagy in CI-AKI

**Figure 8:**
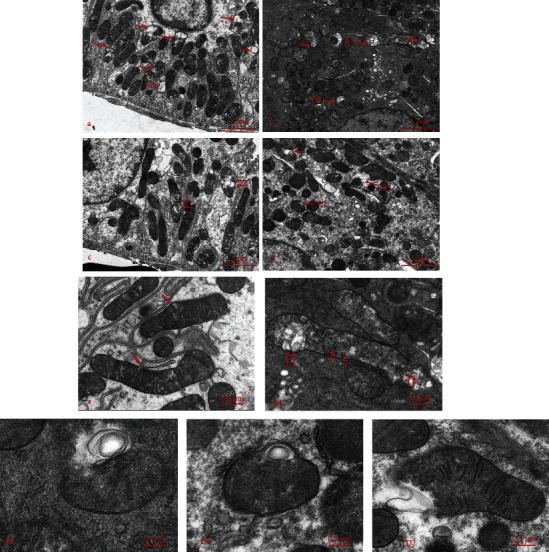
(a, b) TEM of tubular epithelia. Numerous morphologic mitophagosomes are forming after CM injection at 48 h. (a–d) Mitophagosome (red arrows); (e) normal mitochondrion (red arrows). B1, B2, D1, and D2 are magnified on the right. CM: contrast media; TEM: transmission electron microscope.

After the administration of CM for 48 h, we observed mitophagy in the tubular epithelia. Representative images are shown in Figures 8(a) and 8(b). Numerous mitochondria are broken or fused with bubble structures that resemble the lysosomes.

## 4. Discussion

In this article, we established a mouse model of CI-AKI and provided evidence to prove the potential mechanisms of CI-AKI. We proposed three new underlying mechanisms that may exacerbate renal injury: pyroptosis, mitophagy, and inflammation.

Iodinated CM, especially of high osmolality, has higher different viscosities and osmolalities than body fluids [4] [8] [9] [10]. The loading of CM in the kidney affects water movement, perfusion, and diffusion, and this alteration of H_2_O movement can be detected with DTI. Water molecule movement is restricted, and the signal is enhanced after a given CM. In addition, renal hypoxia occurs due to CM taking the place of oxygenated red blood cells through the vessels into the kidney. This alteration can be monitored by BOLD MRI; less-oxygenated tissue weakens the BOLD signal [11]. Both water movement and low oxygen will return to normal with CM metabolism. The physical and chemical features of CM make it toxic to the kidney, and this toxicity can be represented via microscopy with H&E staining. Vacuole formation is the most characteristic change that is rarely found in normal kidneys. Some researchers have noted that these vacuoles are lysosome pinocytosis, and this process is reversible [9]. Other unspecific pathological changes include swollen tubular epithelial cells, shedding, desquamated nuclei, budged lumen, and cast appearance. These microscopic renal morphology changes will return to normal if no CM is further administered. If a patient has renal malfunction worse than the results observed in our experiment, such as diabetic nephropathy [12] or chronic kidney disease (CKD) [13], these renal pathological changes probably will be constant or irreversible.

Previous experiments on the exploration of CI-AKI have mainly focused on excessive ROS (reactive oxygen species) generation and how vascular responses to ROS occur. Many ROS-related molecules [8] have been studied as potential targets of CI-AKI, and we have reviewed potential signaling pathways related to CI-AKI [14]. However, the immune response is always overlooked. Iodinated CM first comes as an alien to the body and then as a medicine that is used in disease diagnosis or therapy. The innate immune response will occur more or less. The immune response protects the body from pathogens and self-damaged cells. In this process, the normal part of the organ (kidney) will also undergo surveillance of the immune system [15]. Our research has also proven that after injection of CM, neutrophils and M1 macrophages are increased in rat kidney [16]. In this study, we chose Ly6G as a neutrophil marker. It was expressed more strong in the cortex glomeruli than in medullar tubules 1 h after CM administration. However, at 48 h, the medullar tubule region expressed the strongest Ly6G. This difference means that neutrophils migrated from the capillary to interstitial tissue around the medullar tubules. The NLRP3 inflammasome is an important component in innate immunity and is a popular research topic in inflammatory diseases. Two kinds of NLRP3 inflammasomes, canonical in leukocytes and noncanonical in the tubular epithelia [15] [17], are found in CI-AKI. In our study, Nlrp3 expression was stronger 12 h after CM injection. Without sustained CM injection, Nlrp3 expression decreases 72 h after the first CM injection. However, we did not distinguish the two kinds of NLRP3 inflammasomes *in vivo*. NGAL is another protein appearing in many kinds of cells, including immune cells, and is one of the best markers of acute kidney injury [18]. NGAL can activate the NF-*κ*B pathway [19], and NF-*κ*B controls the expression of NGAL [19] [20]. It can be induced by various proinflammatory stimuli and expressed by a variety of immune cells, including neutrophils [21], macrophages [22] [23], and dendritic cells [24]. Our study showed that NGAL obviously increased 12 h after CM injection and had not decreased within 72 h, which means that inflammation occurs within 24 h and remained at least until 72 h.

Autophagy is a general regulated process that can remove damaged organelles through lysosomal degradation, and it includes 3 types: macroautophagy, microautophagy, and chaperone-mediated autophagy [25]. Mitophagy is a selective autophagy pathway that targets and destroys mitochondria to maintain mitochondrial quality [26]. Mitophagy has close relations with NF-*κ*B [27], the NLRP3 inflammasome, and HIF-1*α*^28^ [28]. In CI-AKI, tubular epithelial cells are damaged due to increased ROS and CM toxicity and mitochondria undertake a high burden in this process. ROS are a major inducer of mitophagy [29]. Recent studies have found that mitophagy occurs in the tubular epithelium with CI-AKI [30] and that HIF-1*α*-BNIP3-mediated mitophagy in tubular cells plays a protective role through inhibition of apoptosis and ROS overproduction in AKI [28]. Notably, the connection between mitophagy and NLRP3 inflammasome is attracting much attention. Damaged mitochondria in CI-AKI induce the generation of unnecessary mtDNA and mtROS, which activate the NLRP3 inflammasome [31], which in turn is regulated by mitophagy [32] [33] [34]. By eliminating damaged mitochondria, mitophagy suppresses inflammasome activation [35] [36] [37]. Autophagy/mitophagy dysfunction can lead to disease with hyperinflammation and excessive activation of the NLRP3 inflammasome [34] [38] [39] [40]. In macrophages, mitochondrial homeostasis and mitophagy are crucial for the determination of macrophage functional behavior ^30^. M1 macrophages participate in the connection between inflammation and mitophagy [41]. CM-induced hypoxia (increased HIF-1*α*) may induce increased BNIP3L/NIX-dependent mitophagy in proinflammatory macrophages [42]. The balanced activation of the inflammasome-mitophagy pathway may contribute to protecting host normal immunity function and the prevention of harmful inflammatory responses [26].

The apoptosis-related proteins, Bcl2-associated X (BAX) and cytochrome C, were upregulated after CM injection, and the expression of the antiapoptotic protein B-cell lymphoma-2 (Bcl-2) was downregulated. Caspase-8 may be involved in apoptosis, necroptosis, and pyroptosis [43] [44]. Some studies have concluded that caspase-8 is activated within NLRP3 inflammasome signaling platforms [45]. In our experiment, however, the increase in caspase-8 expression was not as high as that of caspase-1 and Nlrp3. This may indicate that pyroptosis, in which Nlrp3 and caspase-1 are involved, dominates tubular epithelial death in CI-AKI. NF-*κ*B plays a major role in the inflammatory pathway, and its expression is increased in CM-administered mice. Its association with NGAL, mitophagy, and Nlrp3 needs further investigation. BNIP3L/Nix is the representative part involved in mitophagy, as we have discussed it earlier, and it was upregulated after CM injection in our experiment. Mitophagy reduces mitochondrial mass as an adaptive response to hypoxia, and it is partially under the control of some stress response pathways [46]. For example, BINP3 is a transcriptional target of hypoxia-induced factor (HIF-1), FoxO3, activated Ras, and p53 while NIX (BNIP3L) is involved in both HIF-1- and p53-regulated pathways [46].

## 5. Conclusion

In conclusion, CI-AKI truly exists, although it is sometimes not easily discernable in clinical practice. fMRI can detect early CI-AKI within 1 h after CM injection. Several mechanisms and pathways are involved in this process. First, renal hypoxia enables apoptosis, and the overproduction of ROS triggers direct cell death. Then, the excess ROS activate inflammatory components such as NF-*κ*B and the NLRP3 inflammasome as well as mitophagy. Moreover, the NLRP3 inflammasome is also involved in CI-AKI and mitophagy may play a role in mitigating kidney injury. Notably, the mitochondrion is one of the key organelles in the tubular epithelium implicated in CI-AKI. Our future research will include *in vitro* experiments to further study the damage to different cell types from CM exposure.

## Figures and Tables

**Table 1:** Parameters and sequences of DTI, BOLD, T1WI, and T2WI. Manufacturer: Bruker BioSpin MRI GmbH; Station name: BioSpec 94/20 USR. DTI: diffusion tensor imaging; BOLD: blood oxygen level-dependent imaging; T1WI: T1-weighted image; T2WI: T2-weighted image.

Parameters	DTI	BOLD	T1WI	T2WI
Number of slices, *n*	195	2340	13	13
Section thickness (mm)	1	1	1	1
Repetition time (ms)	3800	2000	1500	2500
Echo time (ms)	23.5	18	8.5	33
Orientation	Coronal	Coronal	Coronal	Coronal
Bandwidth (hertz per pixel)	2551.020208	2929.6875	348.7723214	183.1054688
Rows and columns	128∗128	128∗128	256∗256	256∗256
Acquisition matrix	0/98/29440/11630	0/128/28160/12590	0/256/20224/28525	0/256/11520/25439
Number of excitations, *n*	1	1	4	4
Acquisition time (s)	152.436	153.133	151.850	151.218
*b*-values (s/mm^2^)	0,1000	—	—	—
Flip angle	90	80	180	180
Breathing protocol	Free	Free	Free	Free

## Data Availability

The data used to support the finding of this study are available from the corresponding author upon request.

## Abbreviations

AKI: Acute kidney injury
BAX: Bcl2-associated X
Bcl-2: B-cell lymphoma-2
BNIP3L/Nix: Bcl2 interacting protein3/Nip3-like protein
BOLD: Blood oxygen level dependent
CI-AKI: Contrast-induced acute kidney injury
CIN: Contrast-induced nephropathy
CMSC: Contrast Media Safety Committee
CM: Contrast media
DAB: Diaminobenzidine
DTI: Diffusion tensor imaging
ELISA: Enzyme-linked immunosorbent assay
ESUR: European Society of Urogenital Radiology
fMRI: Functional magnetic resonance imaging
GAPDH: Glyceraldehyde-3-phosphage dehydrogenase
H&E: Hematoxylin and eosin
HRP: Horseradish peroxidase
IHC: Immunohistochemistry
NF-*κ*B: Nuclear factor kappa-B
NGAL: Neutrophil gelatinase-associated lipocalin
Nlrp3: Nucleotide oligomerization domain-like receptor pyrin 3
PC-AKI: Postcontrast acute kidney injury
ROIs: Regions of interest
ROS: Reactive oxygen species
sCr: Serum creatinine
T1WI: T1-weighted imaging
T2WI: T2-weighted imaging
TEM: Transmission electron microscopy.
